# A Targeted Radionuclide Therapy Facility at a University Teaching Hospital in Singapore

**DOI:** 10.7759/cureus.92308

**Published:** 2025-09-14

**Authors:** Koon Liang Chia, Shao J Ong, Michael Ong, Michael Tong, Hoi Yin Loi, Bertrand Ang, Swee Tian Quek

**Affiliations:** 1 Radiology, National University Hospital, Singapore, SGP; 2 Radiology, National University Singapore, Singapore, SGP

**Keywords:** decay tank, facility design, iodine 131, radionuclide therapy, radiopharmaceutical therapy, shielding

## Abstract

High-dose radioactive iodine is used for the ablation of residual thyroid tissue after thyroidectomy. Patients undergoing such treatment should stay at a purpose-built facility to minimise radiation exposure to the patient’s family, staff, and the general public.

We present our facility, supporting equipment, and measured radiation doses to demonstrate the effectiveness of the radiation safety design.

Our facility comprises an anteroom and two single rooms with ensuite toilets and inter-room shielding. Lead shielding thickness ranges from 5 mm to 25 mm. The emergency shower, hand-foot contamination monitor, survey meter, radiation spill kit, and personal protective equipment are sited in the anteroom. Urine from the hot toilets is collected in dedicated decay tanks before discharge into the main sewage. A high-efficiency particulate air (HEPA) filter-fitted ventilation system with 12 air changes per hour is used to reduce the presence of airborne particulate matter. Radiation detectors are installed directly above the patient beds to enable remote measurement of patient dose rates.

Contaminated items are divided into linen and waste bags before being brought to a waste storage area for radiation decay. These radiation “hot” bags are stored until their projected date of reaching background radiation, after which they are returned to housekeeping or disposed of appropriately.

The waste storage area, built with 5 mm lead lining on top of existing reinforced concrete walls, is sited below the patient rooms. It houses the decay tanks, lead-lined storage bins, and a freezer for contaminated items.

Area monitors are installed in both the anteroom and in the storage area.

## Introduction

Radioactive iodine (RAI) is used for the ablation of residual thyroid tissue after a thyroidectomy. At our institution, RAI therapy is prescribed as either high or low dose, where high dose refers to prescribed activity above 30 mCi. While low-dose treatment is administered on an outpatient basis, high-dose therapy requires an appropriately designed area for the patient to stay while their radiation levels drop to acceptable levels [1, 2]. As the bulk of administered RAI is excreted through bodily fluids [3], the amount of radioactive contamination generated can be significant. Although there are numerous publications on best practices and recommendations on radiation safety and facility design for targeted radionuclide therapy (TRT) [4-6], there is a scarcity of literature that describes constructed facilities to serve as a reference for building planners and radiation protection staff.

Groth recommended the use of 190 mm concrete walls [7], and Vilasdechanon *et al* presented a five-bed facility for RAI therapy at Suandok Hospital, Chiang Mai, Thailand [8]. In land-scarce Singapore, floor space is very limited, which restricts the use of concrete as a shielding material and the overall size of the facility. We present a compact yet safe configuration at a university teaching hospital in Singapore, the equipment and workflows for radiation safety, and measured radiation doses to demonstrate its effectiveness. This work was previously partially presented at the IUPESM World Congress on Medical Physics and Biomedical Engineering in 2022.

## Technical report

Facility design

Our targeted radionuclide therapy facility is sited within a University Teaching Hospital on the western side of Singapore. It was constructed within a hospital extension wing that was originally built in 1996. It was designed to accommodate up to four patients per week for radioiodine targeted radionuclide therapy. 

The facility comprises two separate areas: a patient suite and a storage bunker for radiation-contaminated material. The vast majority of patients stay for three days and two nights, with a maximum facility capacity of four patients per week. Admitted patients are typically adults, medically stable, and activities of daily living (ADL) compliant.

Patient suite

The patient suite was constructed by retrofitting the extension wing of a day surgery ward located two floors above the basement. Of the four external areas directly around the suite, two are of low occupancy factor (a car park and an air-conditioning plant), while the other two are of high occupancy factor (a public corridor and one end of the day surgery ward). The floor directly above the facility is an outpatient clinic, and the floor directly below the facility is the medical record office and store.

The suite itself comprises an anteroom and two patient rooms, each with an ensuite toilet. An emergency shower, a tap and basin, storage cabinets with personal protective equipment (PPE) and a radiation spill kit, a hand-and-foot contamination monitor (HFM), and a Geiger-Muller (GM) pancake survey meter were made available in the anteroom.

The patient rooms and toilets are approximately 15 and 4 square meters, respectively. To reduce the amount of and risk of exposure to airborne RAI particulate contaminants, climate controls were installed with the ambient temperature set at a comfortable 22.5°C and relative humidity of 65%. A high-efficiency particulate air (HEPA) filter-fitted ventilation system with an air flow rate of 12 air changes/hour was also installed. In the toilets, stainless steel toilet bowls capture and channel urine to large-volume decay tanks in the bunker.

Both anteroom and patient rooms, as supervised and controlled areas, respectively, are restricted areas via card access only for trained nursing, physicist, nuclear medicine, and housekeeping staff. The sliding door between the anteroom and the day surgery ward, as well as both treatment room doors, are secured using card access-only magnetic locks. This prevents patients from wandering out into the ward and coming into contact with the general public. Emergency exits are built into the locking mechanisms via break-glass panels and a failsafe that automatically disengages the magnets in the event of an interruption to electrical supply. Nursing call bells are provided for in both rooms.

Objects that are regularly touched by the patients, or with porous or permeable surfaces, e.g., intercom grilles, wooden tables, TV remote controls, etc., are wrapped in food-grade cling film to facilitate cleaning and to prevent contamination from accumulating and persisting. Additionally, the HFM detectors are also wrapped in cling film to allow for continued normal operation in the event of contamination reaching the detector surfaces.

Radiation shielding

**Figure 1:**
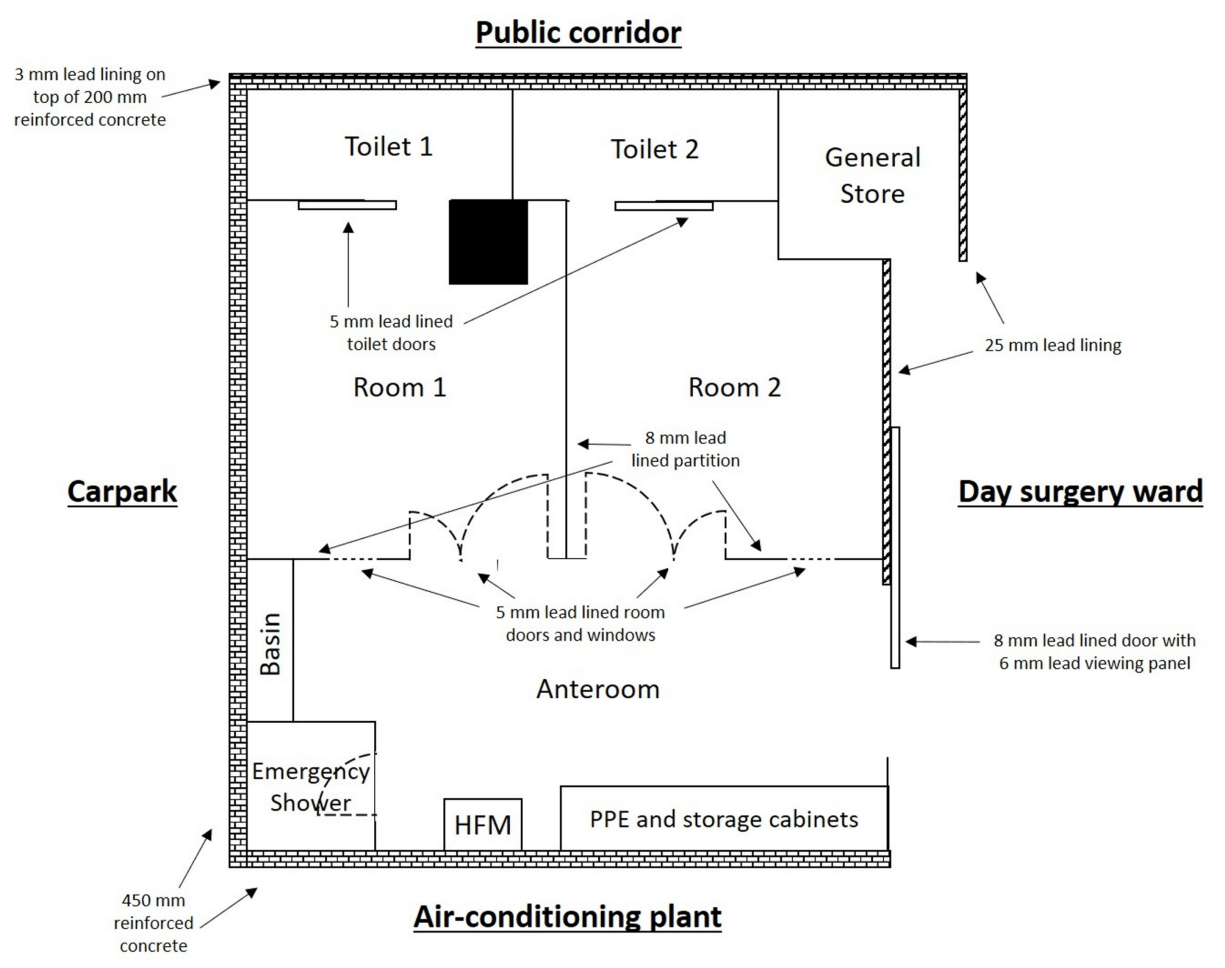
Schematic diagram illustrating the shielding around the anteroom and patient rooms. Unannotated walls are constructed of rebar-reinforced concrete with a thickness of 200 mm. Remote monitoring can be carried out from the nursing station. HFM: hand-and-foot contamination monitor; PPE: personal protective equipment Image credits: Koon Liang Chia. Figure is not drawn to scale.

The periphery walls of the patient suite facing the low-occupancy areas were not further reinforced, as the existing 450 mm reinforced concrete structure was assessed to provide adequate radiation shielding. The floor thickness between the levels is 300 mm of steel rebar-reinforced concrete. The wall facing the ward was topped up with 25 mm lead lining, while 3 mm lead lining was installed on the existing concrete wall between the ensuite toilets and the public corridor. The sliding door entrance to the anteroom and the partition between the patient rooms have 8 mm lead lining, while the room and toilet doors have 5 mm. Windows of 5 mm lead equivalent thickness and intercom systems built next to each room door significantly reduce the radiation exposure to clinicians and nurses when they communicate with patients. A wheelchair bay sited just beyond the 25 mm lead-lined wall helps to maintain at least a 1.5 m distance for staff, patients, and the general public from the wall (Figure 1).

Radiation administration and monitoring

Radioactive iodine is administered orally via capsule within the patient suite by a trained nuclear medicine technician. The choice of capsule oral administration is to reduce the risk of accidental liquid spillage during transport and administration. 

**Figure 2:**
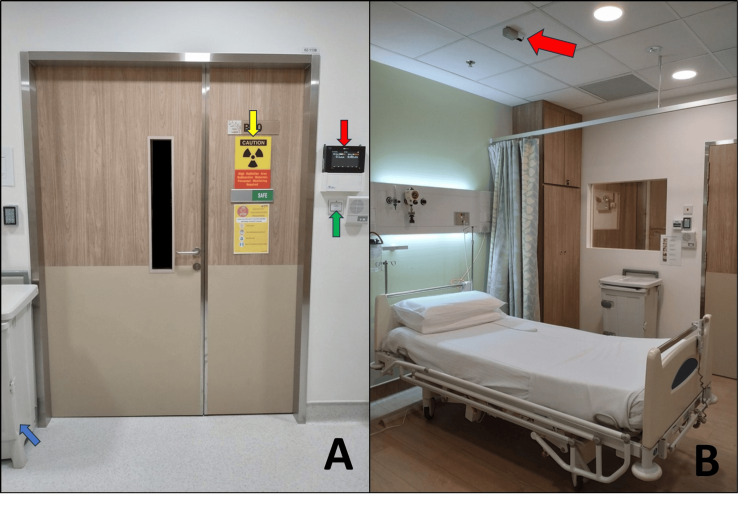
Supporting equipment outside the patient room (A) and Geiger-Müller detector setup inside the room after housekeeping (B). (A) Supporting equipment outside the patient room includes an area monitor (red arrow), radiation warning signage (yellow arrow), magnetic door lock (green arrow), and personal protective equipment bin (blue arrow). (B) The patient room after a completed housekeeping cycle. The Geiger-Müller detector above the bed (red arrow) is distance-corrected to measure the dose rate at 1 m from the patient’s neck.

To further reduce radiation exposure to staff during patients’ stay, a remote monitoring system was installed. This consisted of internet protocol cameras and radiation detectors integrated into a consolidated display on a workstation that is located just outside the anteroom for nurses to remotely monitor patients. Area monitors were positioned just outside each room door, while additional GM detectors were mounted directly above the patient beds to measure the radiation level from the patient (Figure 2). The latter is distance corrected by software to reflect the dose rate at 1 m above the bed around the patient’s neck area and replaces the traditional approach of having staff manually measure the patient's dose rate and assess if the discharge criteria have been met. This remote monitoring system allows for a reduction in radiation dose to staff without compromising patients’ wellbeing and standard of care.

Storage bunker

The bunker is sited approximately underneath the patient suite in a far corner of the basement carpark and houses the decay tanks and lead-lined storage lockers. Lead-lined decay tanks hold the highly radioactive liquid waste, which undergoes radiation decay until it reaches background levels before eventual discharge into sewage. This is especially important in Singapore because wastewater may be captured to be recycled and purified into potable water [9]. To the best of our knowledge, the water purification process is not designed to filter out 131I, and because 131I has a relatively long half-life of 8.04 days, the delay and decay process serves as both radiation protection to the environment as well as to the general public. The decay tanks are located in a restricted area underneath the patient rooms to allow for waste to flow via gravity instead of pumps, thereby reducing the risk of transport failure in the event of mechanical or electrical disruptions. Several redundancies were built into the tank system to reduce the risk of waste overflow, such as battery-powered backup volume monitors, automated software controls to change the active filling tank, and a dilution chamber for mixing the effluent with raw water before discharge into the main sewage.

The lead-lined storage lockers hold contaminated material, either linen or waste, before they are returned to housekeeping or sent for disposal. To conveniently view the storage distribution of the contaminated material, an in-house system was created using craft magnetic sheets and off-the-shelf magnetic labels to display the stored bags’ identification numbers above each locker. The walls facing the accessible carpark area are reinforced with 5 mm lead lining. To constantly measure the amount of radiation in the bunker, a GM area monitor was mounted above the storage lockers; additional GM probes were installed at the bottom of the decay tanks.

Exiting rooms

**Figure 3:**
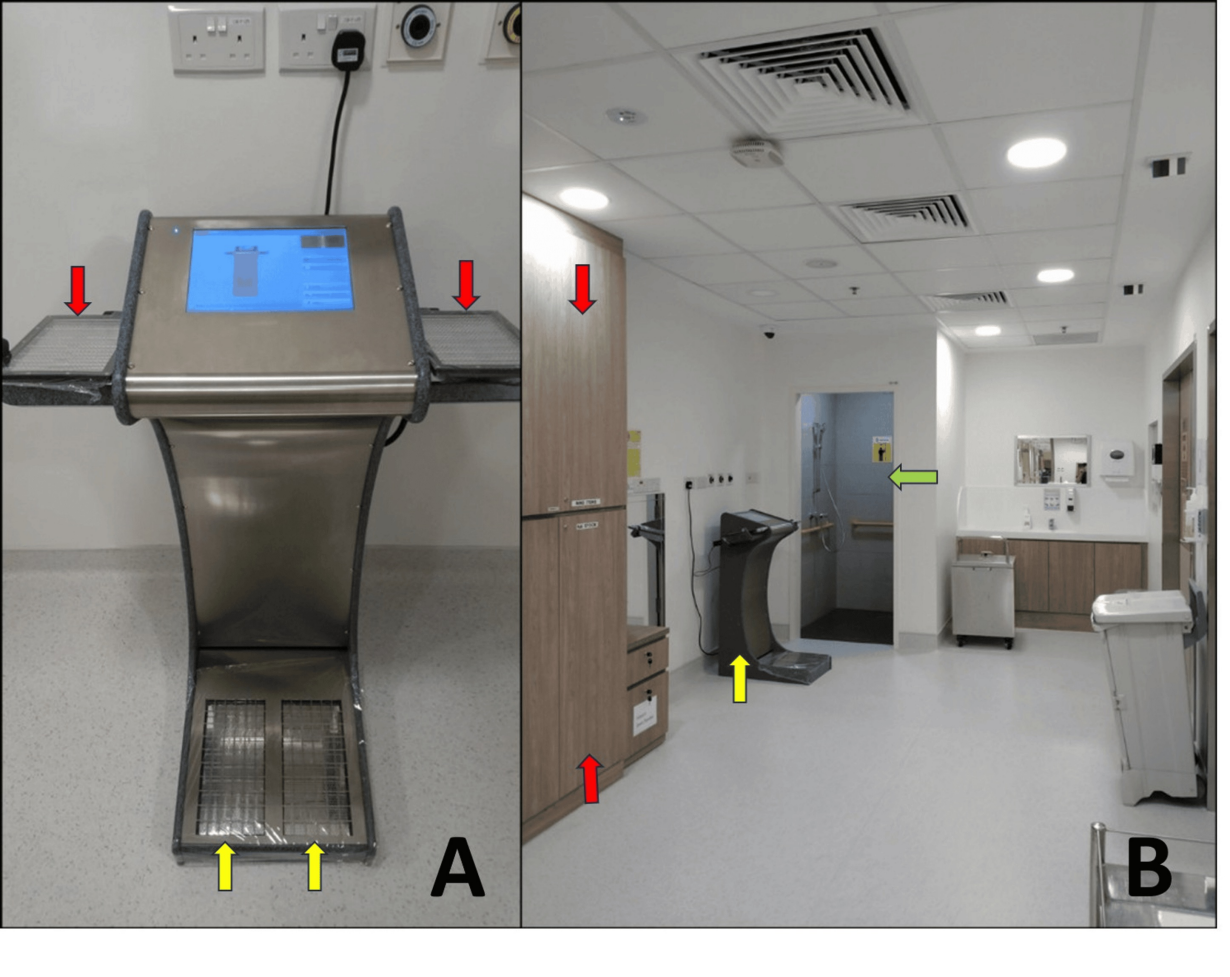
Hand-and-foot contamination monitor (A) and anteroom with storage lockers, supporting equipment, and emergency shower (B). (A) Hand-and-foot contamination monitor. After doffing PPE, staff are required to survey both hands (red arrow) and both feet (yellow arrow) before performing hand hygiene. (B) The anteroom contains storage lockers (red arrow), supporting equipment (yellow), and an emergency shower (green arrow).

Full PPE (hair cover, surgical mask, gown, double gloves, and shoe cover) is donned by nurses before they enter the rooms to take patients’ vitals and to serve meals. Upon exiting, PPE must be doffed in sequence, starting with the outer pair of gloves, then head to toe, and ending with the inner pair of gloves. Before practicing hand hygiene, staff then use the HFM to check for residual contamination (Figure 3). There have been instances where contamination was detected on shoe soles. Decontamination efforts were carried out by using a soft cloth moistened with water, or isopropyl alcohol 70% wipes, to repeatedly wipe the contaminated area in a single direction until a calibrated survey meter measured a dose-rate of less than 1 μSv/hr.

Decontamination

Room decontamination is carried out by the medical physicist to remove grossly contaminated items. The objective of decontamination is not to be exhaustive, as the room would again be contaminated during the next patient stay, but to reduce the radiation exposure to housekeeping staff, who have to change linen and refresh the room and toilet, and to reduce the risk of spreading contamination when nurses bring patients into the room to orientate them prior to their stay. Contaminated items are separated into linen and waste bags, and a GM pancake survey meter is used to measure the maximum surface dose rate. Each bag is then labelled with an identification number, the isotope, and the maximum surface dose rate before being brought down in a lead-lined trolley to the bunker for storage and decay. Soiled linen or waste, such as bedsheets with vomitus or diapers with faeces, is stored in a lead-lined freezer to slow the growth of bacteria and stench.

On the completion of decontamination, the medical physicist changes the radiation warning signage from “Radiation” to “Safe”. All staff are trained to recognize that “Radiation” indicates a high dose rate and/or risk of exposure to contamination, and “Safe” indicates that the area is free of any radiation contamination. Housekeeping staff will not enter the patient rooms when the radiation warning signage shows “Radiation”; they have escalated housekeeping requests to the medical physicists for clarification on several occasions. Similarly, nurses will turn away and reschedule routine maintenance events. The use of warning signage and appropriate training is an efficient and effective method of communicating radiation hazards asynchronously across specialist and non-radiation specialist job groups.

Housekeeping

**Figure 4:**
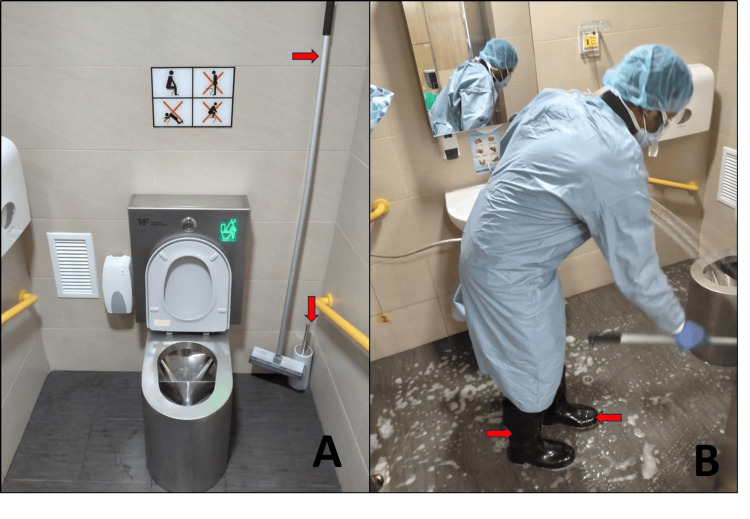
Stainless-steel toilet with separate compartments (A) and housekeeping staff using protective equipment during cleaning (B). (A) Stainless-steel toilet bowl with separate compartments for liquid and solid waste. Dedicated cleaning equipment can be seen in the background (red arrow). (B) During cleaning, housekeeping staff are required to wear full personal protective equipment, including rubber boots (red arrow), to prevent external contamination from wastewater.

Local rules were developed specifically for the cleaning of the toilet, as it is the area with the largest amount of radioactive contamination. We follow a cardinal rule of having dedicated cleaning equipment for use only in the room toilets and not to circulate brushes and mops among the general housekeeping supply. All housekeeping staff don PPE and waterproof boots prior to entering the toilet (the boots are kept in a stainless steel storage cabinet in the room) in order to prevent radioactive wastewater from coming into contact with their skin (Figure 4). The cleaning cloths used to wipe down the mirrors and toilet seats are brought out and marked as radiation-contaminated waste. Cling wraps, for both room items and the HFM, are replaced at the end of the housekeeping cycle.

Storage management

**Figure 5:**
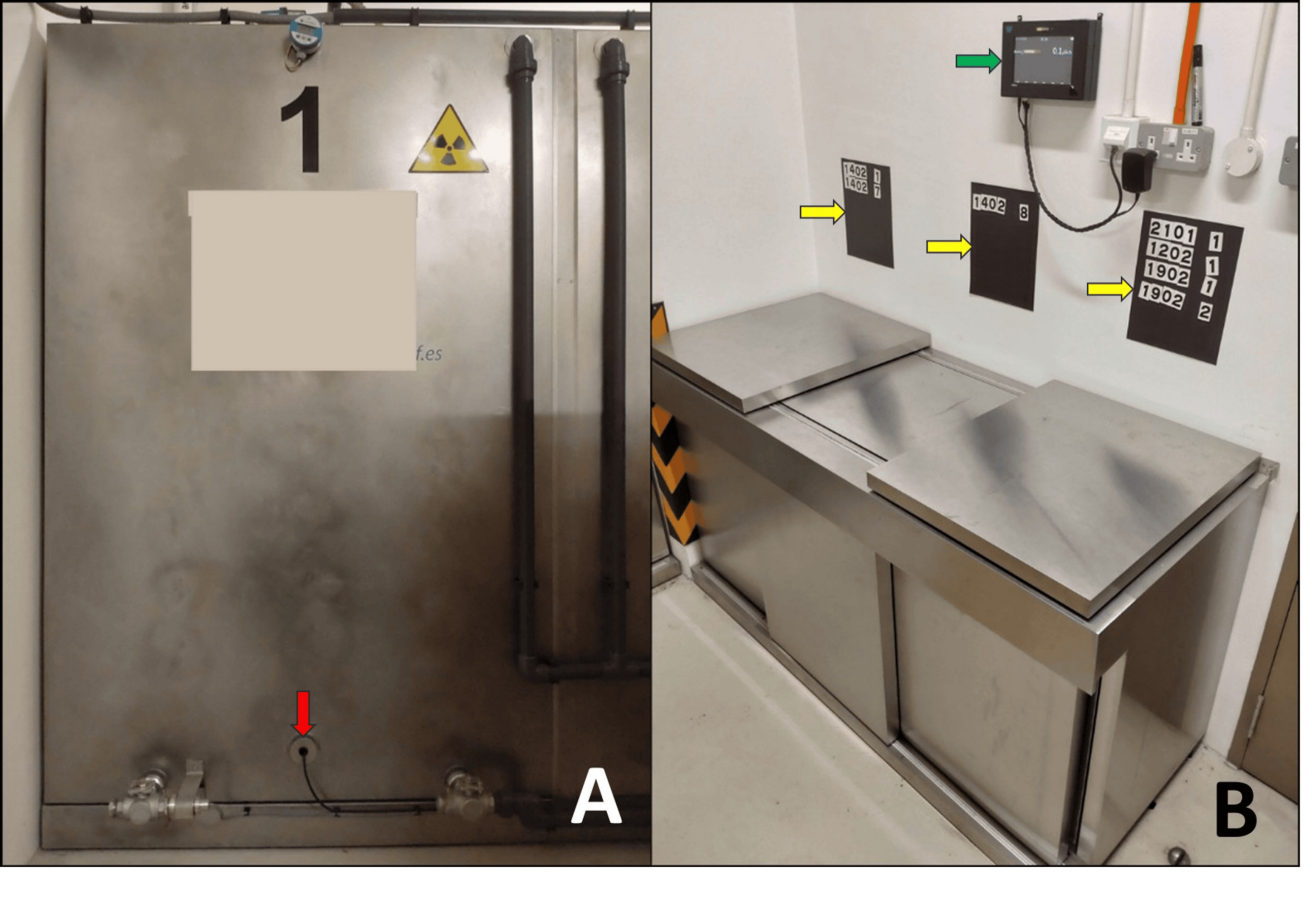
Decay tank with monitoring equipment (A) and lead-lined lockers with area monitor and display system (B). (A) One of three 3000 litre decay tanks, with a backup volume monitor and Geiger-Muller probe (red arrow). (B) Lead-lined lockers used for storage of contaminated material, with an area monitor (green arrow) and an in-house display system (yellow arrows) mounted above.

To efficiently manage the limited storage space, we dispose of contaminated waste or return linen to housekeeping within two weeks of reaching background doserates. Instead of manually surveying all bags every few weeks, which involves more time spent and radiation exposure to residual contamination, the medical physicist forward-estimates when each bag will reach background. Once ready, the display system above each storage locker is referred to for retrieving the specific bag for disposal. The maximum surface dose rate, estimated time to decay, and date of disposal/return are recorded in a digital log sheet for oversight and end-to-end tracking of all contaminated material from generation to storage to disposal (Figure 5).

## Discussion

Following the commissioning of the facility, we followed up with measurements and estimation of radiation exposure to staff and the general public. Radiation exposure to staff was expected to be low or negligible. Several radiation measurements were made to gauge the effectiveness of the facility design and workflows.

First, exposure to the general public was estimated via readings measured at the surface of the 25 mm lead-lined wall to the ward using a pancake GM survey meter (ASM-993, Fluke Biomedical, Cleveland, OH, USA) 30 minutes after administration of 150 mCi RAI; all measurements were no more than 2 μSv/hr. Second, the exposure to housekeeping staff (non-radiation workers) was measured using calibrated electronic personal dosimeters (EPDs) (DMC 3000, Mirion Technologies, Atlanta, GA, USA) that were placed in their uniform pockets located at waist height. In a typical 30-minute housekeeping cycle, all EPD readings were negligible.

Third, the maximal bi-monthly dose to nursing, physicists, and nuclear medicine staff was estimated at 1.44 mSv based on the highest historical EPD reading of 30 μSv from a 15-minute close-contact exposure to a nurse. All staff, including housekeeping, are rotated to spread out the dose burdens and are trained to plan ahead to reduce the time spent in the patient rooms. The total dose in a year to radiation workers does not exceed the regulatory limit of 20 mSv in Singapore and is in agreement with other institutions with I131 radionuclide therapy services [10,11], demonstrating the effectiveness of our facility design.

## Conclusions

In summary, we present our compact, purpose-built facility for high-dose RAI patients. The installation of lead-lined walls, a monitoring system, and site-specific workflows helps to reduce the amount of radiation exposure to clinicians, nurses, and housekeeping staff. Radiation safety for the environmental and general public was also achieved by the use of a decay tank system. Radiation doses, both measured and estimated, were low and demonstrate the effectiveness of the facility design and workflow processes.
